# Gestational Trophoblastic Disease Presents as an Ectopic Tubal Pregnancy, a Rare Entity

**DOI:** 10.1155/2019/7153170

**Published:** 2019-08-06

**Authors:** Haneen Al-Maghrabi, Daniyah Saleh, Abdelrazak Meliti

**Affiliations:** ^1^Department of Anatomic Pathology, King Faisal Specialist Hospital and Research Center, Jeddah, Saudi Arabia; ^2^Department of Laboratory Medicine & Pathology, University of Alberta, Edmonton, AB, Canada

## Abstract

Ectopic molar pregnancy is an uncommon event in clinical practice. In this paper, we report a case of ectopic complete molar pregnancy in a 39-year-old lady who presented to the emergency department with lower abdominal pain, abdominal distention, and low-grade fever. Based on radiological and laboratory investigations, the differential diagnosis included ruptured ectopic pregnancy versus metastatic diseases. Ectopic hydatidiform molar pregnancies can occur at any extrauterine pelvic sites, yet more frequently affecting fallopian tubes. The histopathological examination remains the gold standard for the diagnosis.

## 1. Introduction

Hydatidiform moles are placental abnormalities with distinct genetic disorders that cause variable trophoblastic disease and hydropic villous changes [1]. Complete and partial moles are two different morphologic subtypes of hydatidiform moles, based on their presenting clinical symptoms, genetic abnormality, morphologic morphology, and patient outcome [2]. The distinction between the two entities is essential for clinical management and patient outcome. Complete hydatidiform mole (CHM), likewise in the present case, is purely androgenic in origin. Over 90% are 46XX, arising from chromosomal duplication of a haploid sperm with an inactive egg or an ovum with no maternal chromosome. Around 10% of cases are 46XY, as a result of fertilization of two sperms into an empty ovum. Rare cases of CHM are tetraploid rather than diploid and are also androgenic in origin. Patients with CHM present with vaginal bleeding, abnormal ultrasound findings, or history of missed abortion. The incidence of ectopic pregnancy is 20 in 1000 and that of hydatidiform molar pregnancy is 1 in 500-1000; nevertheless, the diagnosis of an ectopic molar pregnancy is uncommon with an estimated incidence approximately 1.5 in every 1,000,000 pregnancies [3]. One hundred thirty-two cases of ectopic molar pregnancy of all body sites have been described in a sizable study (1986-2004) [4].

## 2. Case Report

**Figure 1:**
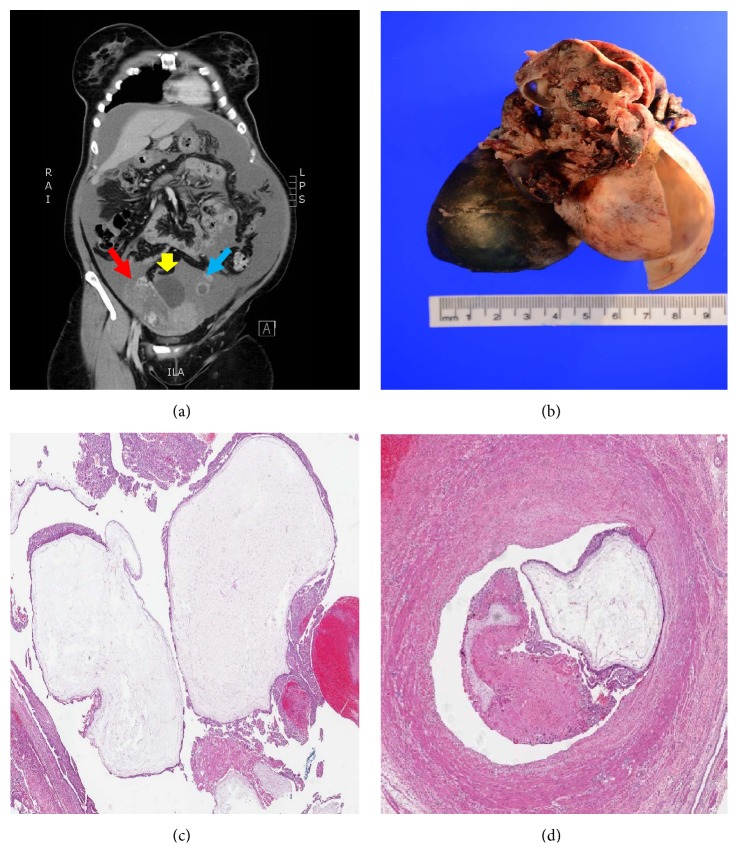
(a) CT scan without contrast revealed large right complex adnexal mass (red arrow), with the attached cyst (yellow arrow) and the left ovary (blue arrow). The blue arrow shows the left ovary. (b) Gross photo shows ruptured fallopian tube with placental-like tissue and adjacent cyst. (c) Hematoxylin and eosin (H&E) histopathology examination demonstrates dilated villi with cistern and circumferential trophoblastic proliferation (H&E; 1x). (d) Vascular invasion (H&E; 1x).

A 39-year-old lady presented with progressive continuous lower abdominal pain, abdominal distention, and low-grade fever for three days. She had four previous cesarean sections; the last one was four years ago. She was not known to have any chronic disease or allergy history. On admission, the patient presented to the emergency department in a state of low blood pressure and received immediate medical treatment. Laboratory workup showed *β*-human chorionic gonadotropin (*β*-hCG) level at 110.766 mIU/mL. Ultrasonography, done at the time of admission, revealed right adnexal heterogeneous complex mass (7 x 5 x 5 cm) and an adjacent right ovarian cyst (8 x 7 x 5 cm) with free fluid detected in the abdomen and pelvis. There was no intrauterine gestational sac. The patient underwent an immediate computerized tomography (CT scan) to assess the extent of the disease which showed large right adnexal mass with abdominal ascites and numerous peritoneal lesions (Figure 1(a)). Once the patient stabilized hemodynamically, she underwent an exploratory laparotomy. The patient consented for definitive treatment if an unexpected underlying malignancy was detected. Intraoperatively, 4.8 liters of blood was suctioned out from the peritoneal cavity. On the right abdominopelvic side, there was clinical evidence of ruptured ectopic tubal pregnancy as well as an adjacent right ovarian cyst with a smooth outer surface. However, there was no evidence of peritoneal carcinomatosis. The patient tolerated the procedure well and transferred to the surgical intensive care unit (ICU) in a stable condition. A right salpingo-oophorectomy was performed and was sent for histopathology for further evaluation and examination. The ovary measures 10 x 7.5 x 4 cm with a cystic lesion (2.2 cm in greatest dimension).

The outer surface of the cyst was smooth, and upon opening the cyst, it yielded clear to yellow fluid. The attached hemorrhagic and ruptured fallopian tube (3 x 2.7 cm) exhibits multiple small vesicles and fragments of placental-like tissue in the vicinity of the perforated areas (Figure 1(b)). Microscopic examination revealed diffuse villous dilatation with substantial hydropic changes. Circumferential trophoblastic proliferation with cistern formation was readily appreciated (Figure 1(c)). Besides, cytological atypia, mitosis, apoptotic debris, and vascular invasion were identified (Figure 1(d)). The morphological features were classic for complete hydatidiform mole, interestingly enough, arising in the background of ectopic (tubal) pregnancy. Apart from the molar pregnancy, the examination of the juxtaposed ovarian cyst was that of a benign serous cystadenoma. The patient was followed up strictly with serial blood workup, mainly monitoring of *β*-hCG levels, which showed a rapid drop to less than one mIU/mL over the subsequent months.

## 3. Discussion

Tubal hydatidiform molar pregnancy is an uncommon event with few cases reported in the literature [4]. The median maternal age for gestational trophoblastic disease (GTD) is 31 years old, and the median gestational age is ten weeks [5]. GTD can precede any type of conception including term pregnancy, molar pregnancy, abortion, and ectopic tubal pregnancy [6]. As discussed above, the GTD occurs due to genetic aberrations which lead to placental and trophoblastic malformations, with histologic features of villous swelling and hydropic changes. It is commonly developed within the uterine cavity, but in rare cases can occur as an ectopic tubal pregnancy. Burton et al. and colleagues studied the hydatidiform mole in early tubal ectopic pregnancy for ten years and concluded that it is an overall a rare diagnosis which is overlooked and overdiagnosed by pathologists [7]. Clinical presentation varies from abdominal pain to vaginal bleeding. Ultrasonography studies demonstrate solid and partially cystic mass with vesicular patterns of multiple echoes and holes within placental tissue. The distinction between complete and partial mole is important, and the histopathological examination is the critical distinguisher. Strict criteria should be used to diagnose complete and partial moles. These include circumferential trophoblastic proliferation, hydropic changes, scalloped villi, and stromal karyorrhexis. The use of DNA flow cytometry analysis can help to differentiate. Extravillous trophoblastic proliferation can be more florid than the usual proliferation seen in the typical uterine product of conception. Laparoscopic evacuation with/without salpingectomy is considered the primary choice of treatment in ectopic pregnancies [4]. The *β*-hCG level is monitored more frequently in cases of ectopic pregnancy. Continuous titers follow-up is helpful to diagnose persistent ectopic pregnancy and to exclude malignant trophoblastic transformation [8]. The *β*-hCG serum levels continued to show a substantial drop postoperatively.

Clinical follow-up showed no evidence of recurrent, persistent, or metastatic disease. Complete hydatidiform moles demonstrate negative p57KIP2 immunostaining in cytotrophoblasts and villous stromal cells, as opposed to positive immunoreactivity seen in partial moles [9, 10].

## 4. Conclusion

A case of gestational trophoblastic disease presented clinically as an ectopic tubal pregnancy; interestingly, the histopathologic examination demonstrates classic features of complete hydatidiform mole. The distinction between partial and complete hydatidiform moles is crucial for clinical management and patient outcome. Continuous monitoring of the *β*-hCG levels is vital in postsurgical management.
